# Retrospective single-centre analysis of IgG4-related disease patient population and treatment outcomes between 2007 and 2017

**DOI:** 10.1093/rap/rkz014

**Published:** 2019-05-06

**Authors:** Chan Mi Lee, Mohamed Alalwani, Richard A Prayson, Carmen E Gota

**Affiliations:** 1 The Education Institute, Cleveland Clinic Lerner College of Medicine of Case Western Reserve University; 2 Department of Rheumatologic and Immunologic Disease, Orthopaedic and Rheumatologic Institute, Cleveland Clinic; 3 Department of Anatomic Pathology, Cleveland Clinic, Cleveland, OH, USA

**Keywords:** IgG4-related disease, medical treatment, surgical treatment, outcomes

## Abstract

**Objective:**

The aim was to gain a better understanding of the prevalence, organ involvement, clinical characteristics and long-term outcomes of medical and surgical treatments of IgG4-related disease (IgG4-RD).

**Methods:**

Query of the Cleveland Clinic pathology database for IgG4 plasma cell staining between 2007 and 2017 yielded 1481 results, of which 57 cases were identified as highly likely (*n* = 28; 49%) or probable (*n* = 29; 51%) IgG4-RD by histopathological criteria and IgG4 serum concentrations. Patient demographics, type of treatment and outcomes were retrieved from medical records. Patients were designated as being in remission if indicated in the chart and/or symptom- and objective finding-free for >6 months, relapsed if symptoms/findings recurred after remission, active if no remission was achieved during follow-up, and as unable to determine if the duration of follow-up was <60 days or they were lost to follow-up.

**Results:**

Of all patients who met the IgG4 staining criteria (*n* = 119), half (*n* = 57) satisfied the IgG4-RD histopathological criteria; 63% were males, age 57.9 ± 14.8 years. The average follow-up was 2.7 ± 2.2 years. The pancreas was the most affected organ in our cohort (26.4%). Almost half of the patients (45.6%; *n* = 26) were managed surgically, 21.1% (*n* = 12) medically, and 24.6% (*n* = 14) received both types of treatment. Medical treatment included prednisone (45.6%), MTX (5.3%), AZA (7%) and rituximab (8.8%). Remission was achieved by 77% of patients receiving surgical, 67% receiving medical and 72% receiving both treatments.

**Conclusion:**

A histological diagnosis of IgG4-RD could be made in half of the patients who had IgG4^+^ plasma cells ≥10/high-power field or IgG4^+^/IgG^+^ ratio >40%. In our cohort, surgical treatment compared with medical treatment had a higher proportion of remission according to our outcome classification.

**Key messages**

- IgG4-related disease is rare, with the pancreas being the most affected organ.
- Histopathological criteria were met in half of cases meeting IgG4^+^ plasma cell ≥10/high-power field or IgG4^+^/IgG^+^ ratio >40%.
- In some patients with IgG4-related disease, surgical intervention may be more effective than medical treatment.

## Introduction

IgG4-related disease (IgG4-RD) is a rare, multisystem fibro-inflammatory condition, characterized by organ mass lesions, IgG4^+^ lymphoplasmacytic infiltrate and storiform fibrosis [1–4]. Early detection of IgG4-RD is necessary to prevent organ damage and functional impairment [5, 6] by allowing prompt intervention. Evidence suggests that IgG4-RD may be responsive to immunosuppressive therapy, including glucocorticoid and rituximab [7, 8]. IgG4-RD can also mimic various malignant, infectious and inflammatory conditions [8, 9], requiring better ways to distinguish it from other conditions.

However, as a recently recognized disease, ACR-approved criteria for IgG4-RD classification are still pending. There have been several publications on consensus guidelines for the diagnosis or classification of IgG4-RD [2, 10–13], but clinical characteristics and IgG4^+^ plasma cell cut-offs vary by tissue type and affected organs [12]. For example, IgG4-RD has been most studied in type 1 autoimmune pancreatitis and generally thought to affect more men >55 years old [14, 15], but this gender propensity has not been true for lesions of the head and neck [14]. Additionally, there has been no unifying definition of treatment outcomes, making it challenging to cross-reference conclusions on treatment outcomes from different studies [14–17]. The current treatments for IgG4-RD are still empirical [7]. Glucocorticoids alone show a good initial response, but this approach is plagued by high rates of relapse [18].

Here, we conducted a retrospective cohort study of patients with IgG4-RD at Cleveland Clinic from 2007 to 2017, identified by histopathology, to gain a better understanding of its prevalence, patient population, organ involvement, histopathological characteristics and long-term outcomes of treatments.

## Patients and methods

### Study population and identification of patients

This study was approved by the Cleveland Clinic Institutional Review Board (reference no. 17-367). Patients were identified via a search of the surgical pathology database in Anatomic Pathology at the Cleveland Clinic Foundation between 2007 and 2017, for resected or biopsied pathology specimens that were stained for IgG4. Distinction of pathology reports based on biopsy *vs* surgical specimens was made from the patients’ medical records. Confirmed by the date of the procedure and the date of the pathology report, patients who underwent surgical excision as part of therapy were designated as surgical, and their samples were from their surgical procedure. Patients who underwent only biopsy as part of the diagnostic work-up and who received only medical treatment were classified as medical. Cases were selected based on histopathology, following recently published consensus guidelines for the diagnosis of IgG4-RD [1, 13, 19].

### Classification of IgG4-related disease

**Fig. 1:**
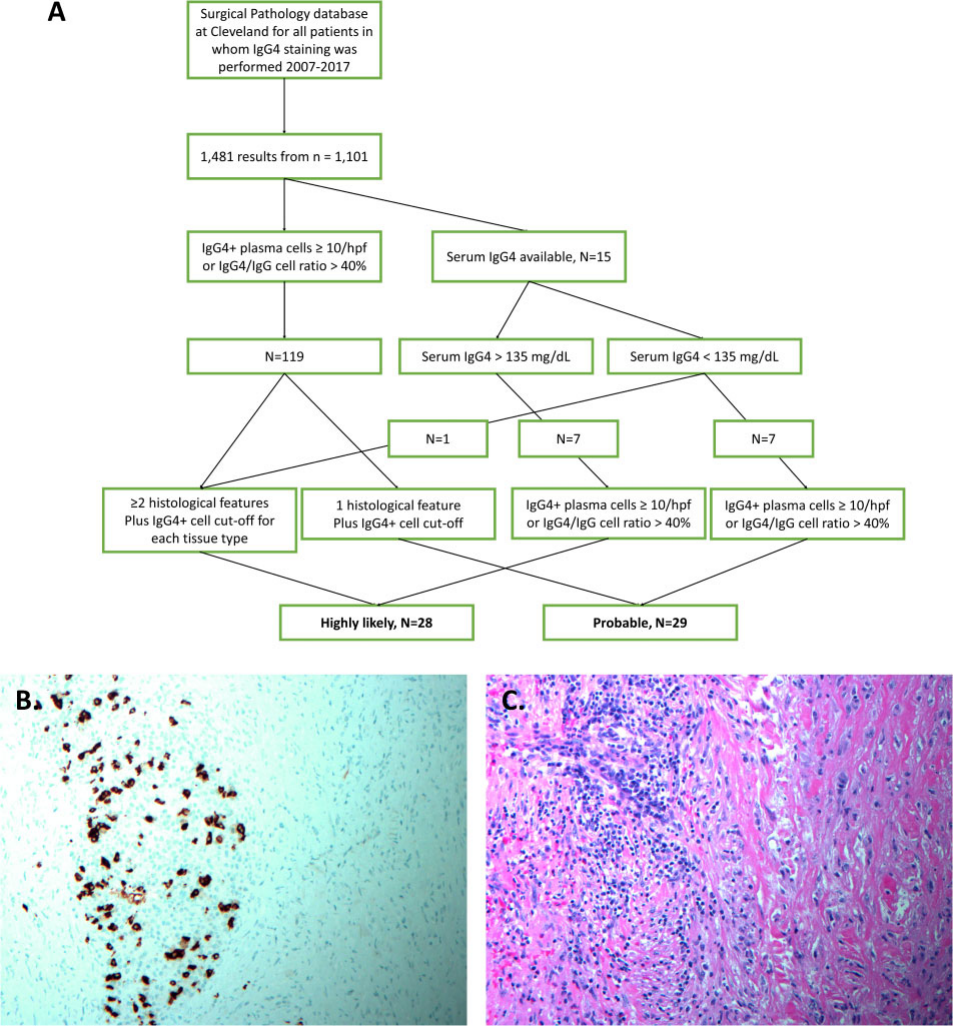
Classification scheme for study population and histopathological analysis from one of the patients showing IgG4^+^ plasma cell staining and histopathology characteristics (**A**) Patients were sorted by both histopathology and the serum IgG4 levels where available. (**B**) Positive immunohistochemical IgG4^+^ staining and up to 29 IgG4^+^ plasma cells/high-power field (hpf) were seen in this patient within the sections of gallbladder wall. (**C**) Histopathological changes showing reactive epithelial changes, marked mural fibrosis with a storiform pattern, and prominent lymphoplasmacytic aggregates extensively involving the subserosal tissue, consistent overall with IgG4-related cholecystitis. This test was developed and its performance characteristics determined by Cleveland Clinic's Robert J. Tomsich Pathology and Laboratory Medicine Institute.

IgG4-RD was classified according to previously published criteria [3, 9, 10] based on the descriptions in the pathology report and availability of IgG4 serum levels (Fig. 1A). If only histopathology was available, cases were defined as highly likely if they had two or more characteristic histological features of dense lymphoplasmacytic infiltrate, fibrosis, obliterative phlebitis plus previously defined IgG4^+^ plasma cell cut-offs for each tissue type [3]; cases with one pathology feature and with sufficient IgG4^+^ plasma cell count were classified as probable. If IgG4 serum levels were available, which was the case for 15 patients, the flow sheet from Yamamoto *et al.* [11] was followed, where IgG4 >135 mg/dl and infiltration of IgG4^+^ plasma cells with IgG4^+^ to IgG^+^ ratio >40% and/or >10 IgG4^+^ plasma cells/high-power field (hpf) were designated highly likely [10, 11]. An example of positive staining results and the histopathological features from a patient included in the study is demonstrated in Fig. 1B and C. Patients with primary diagnoses of B-cell lymphoma, for which the treatment was directed towards the primary diagnosis other than IgG4-RD, were excluded, and those with insufficient information on the pathology report to determine the presence of IgG4-RD were also excluded. With three exceptions, all patients had a diagnosis of IgG4-RD in their medical charts. Three patients were diagnosed with other conditions in addition; one patient was diagnosed with a mediastinal mass with possible Epstein-Barr virus infection, one with pancreatic cancer, and another with granulomatosis with polyangiitis. We included these three patients in our outcomes analysis because they met the histopathological criteria for IgG4-RD.

### Type of treatment and organ involved

We defined medical treatment as use of prednisone, MTX, AZA and rituximab, and recorded the number of medications prescribed for each patient. Surgical treatment was reserved for patients who underwent therapeutic excision of the mass as indicated by the surgery notes and verified by CT scans. Patients who underwent both medical and surgical treatment during the duration of follow-up, such as if the patient was treated medically first before undergoing surgery or received additional medical treatment such as prednisone after surgical removal, were categorized separately as both treatments.

### Outcome measures

Patient age, gender, BMI, pre-existing conditions, presence of allergies, smoking status, alcohol intake, organ involved, type of treatment and outcomes were retrieved from their medical records. Patients were designated as being in remission if indicated in the chart and/or symptom- and objective finding-free for >6 months, relapsed if symptoms or findings recurred after an identified period of remission, active if no remission was achieved during follow-up, and as unable to determine if the duration of follow-up was <60 days or if the patients were lost to follow-up to identify the final outcome. The date of diagnosis for the study was defined as the date of the pathology report that demonstrated the identifying features of IgG4-RD, and the total duration of follow-up was measured from the date of the pathology report indicating IgG4-RD until the last date of a patient visit to the Clinic at the time of chart review. The duration of time until relapse was obtained from the number of days between the date of remission and the best estimate of the date of relapse by a patient visit after previously noted remission.

### Statistical analysis

The statistical analysis was performed using JMP Pro v.13 (Cary, NC, USA). Significance level was set at an α value of 0.05. The χ^2^, Pearson and/or Fisher’s exact test was used to compare categorical variables, and Student’s two-tailed *t*-test was used to compare continuous variables.

## Results

### Number of IgG4-RD patients identified

The search of the Cleveland Clinic pathology database for patients whose biopsy or surgical specimen was ordered an IgG4 staining between 2007 and 2017 identified 1481 pathology cases from 1101 patients. We screened for cases positive for tissue IgG4^+^ plasma cells ≥10/hpf and/or IgG4^+^/IgG^+^ plasma cell ratio >40% (Fig. 1A) based on the comprehensive diagnostic criteria for IgG4-RD, in which a definite diagnosis of IgG4-RD is made if there is demonstration of: (a) organ involvement; (b) serum IgG4 level >135 mg/dl; and (c) >10 IgG4^+^ plasma cells/hpf and IgG4^+^/IgG^+^ plasma cell ratio of ≥ 40% [10]. We found that making a definite diagnosis of IgG4-RD was difficult and thus, instead of definite, we defined our cases as highly likely or probable. Of the 119 positive cases that had tissue IgG4^+^ plasma cells ≥ 10/hpf and/or IgG4^+^/IgG^+^ plasma cell ratio >40%, a total of 57 cases were classified as highly likely (*n* = 28; 49%) or probable IgG4-RD (*n* = 29; 51%), based on histopathological criteria. We used the more inclusive criteria of and/or for IgG4^+^ plasma cells ≥ 10/hpf and/or IgG4^+^/IgG^+^ plasma cell ratio >40%, instead of requiring both conditions, because most pathology reports chose to state either the IgG4^+^ plasma cell count or the IgG4^+^/IgG^+^ plasma cell ratio. Also, not all patients had their serum IgG4 level measured. Of the 119 patients, 15 patients had serum levels of IgG4 available in addition to the histopathology data, and were sorted following the guideline provided by Yamamoto *et al.* [11] to classify them as highly likely or probable (Fig. 1A).

### Baseline characteristics of the study population

Baseline characteristics of highly likely and probable IgG4-RD patients were analysed in comparison, in addition to the whole group (Table 1). Of 57 patients, 63% were male, 57.9 ± 14.8 years old, and BMI 28.9 ± 7.3 kg/m^2^. The racial distribution was 57.9% white and 26.3% African-American (Table 1). A history of allergy was found in 57.9% (*n* = 33) of patients; 54.4% (*n* = 31) of patients had a smoking history, mean 30.1 ± 34.1 pack years, and 45.6% (*n* = 26) of patients had a history of alcohol use (Table 1). We found no statistical differences between highly likely and probable groups with regard to gender (*P *=* *0.4173), age (*P *=* *0.7623), BMI (*P *=* *0.6568) or race (*P *=* *0.1303). The mean duration of follow-up for patient treatment and outcome after identification of IgG4-RD by histopathology was 2.7 ± 2.2 years.

**Table 1:** Demographics of IgG4-realated disease patient population at Cleveland Clinic

		Highly likely (*n* = 28)	Probable (*n* = 29)	Total (*n* = 57)	*P*-value
		Percentage of total (*n*)	Percentage of total (*n*)	Percentage of total (*n*)	
Gender	Male	28 (16)	35 (20)	63 (36)	0.4173
	Female	21 (12)	16 (9)	37 (21)	
BMI	Mean ± s.d. (*n*)	29.4±7.9 (25)	28.5±6.9 (29)	28.9±7.3 (54)	0.6568
Age	Mean ± s.d. (*n*)	58.6±14.8 (28)	57.4±15.0 (29)	58.0±14.8 (57)	0.7623
Race	White	22.8 (13)	35.1 (20)	57.9 (33)	0.1303
	African-American	14.0 (8)	12.3 (7)	26.3 (15)	
	Asian	1.8 (1)	0.0 (0)	1.8 (1)	
	Multiracial	0.0 (0)	1.8 (1)	1.8 (1)	
	Not available	10.5 (6)	1.8 (1)	12.3 (7)	
Alcohol history	Yes	22.8 (13)	22.8 (13)	45.6 (26)	0.2558
	No	22.8 (13)	28.1 (16)	50.9 (29)	
Smoking history	Yes	5.3 (3)	5.3 (3)	10.5 (6)	0.9262
	No	42.1 (24)	45.6 (26)	87.7 (50)	
Allergies	Yes	21.1 (12)	21.1 (12)	42.1 (24)	0.9101
	No	28.1 (16)	29.8 (17)	57.9 (33)	
Duration of follow-up	Years (min–max)	3.1 (0.1–8.6)	2.2 (0–7.1)	2.7 (0–8.6)	0.1335

Numerical variables, such as BMI and age, are presented as the mean ± s.d., with the number of data points that were available indicated in parentheses. The duration of follow-up is presented as the mean value (in years), with the range in parentheses. *P*-values were calculated by two-sample *t*-tests for percentages for categorical variables, and by Student’s *t*-test for numerical variables.

### Organ involvement and histopathological features

**Fig. 2:**
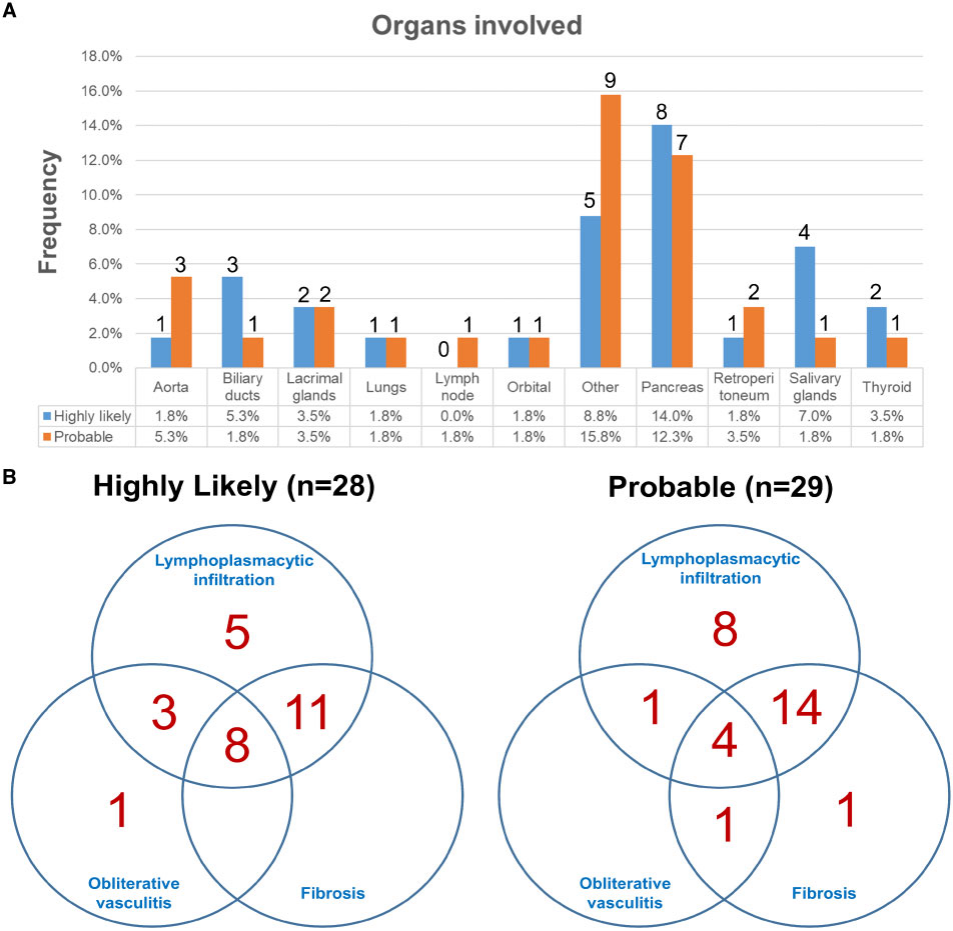
Distribution of patients by organ type and histopathological features (**A**) The proportion of organs involved in patients with highly likely (red) IgG4-related disease (IgG4-RD) or probable (blue) disease. Percentages are the proportion of patients with the particular organ involvement out of the total study sample of *n* = 57 patients. The number of patients with the organ involvement is shown at the top of each bar graph. (**B**) Venn diagram of number of patients with respective histopathological characteristics of IgG4-RD.

Similar to previous IgG4-RD reports, the pancreas was the most affected organ in our cohort (26.4%) (Fig. 2A). Other organs most frequently involved were the pericardium (3 out of 14), gallbladder, liver, rectum, mesentery, terminal ileum, thymus, breast and kidney. On histopathological examination, 94.7% of patients had tissue lymphoplasmacytic infiltration, 68.4% fibrosis and 31.6% obliterative phlebitis; 41 patients (71.9%) had at least two, and only 12 patients (21.1%) met all three histopathological criteria (Table 2; Fig. 2B). Eosinophilic infiltration, which is a recognized feature of IgG4-RD, but not part of the current classification system, was present in eight patients (14%). The average IgG4 plasma cell count was 49.7 ± 36.2/hpf. Of seven patients who had serum IgG4 >135 mg/dl, four demonstrated two or more histological features required for inclusion in the highly likely category.

**Table 2:** Histopathology analysis and IgG4 count derived from pathology report

		Highly likely (*n* = 28)	Probable (*n* = 29)	Total (*n* = 57)	*P*-value
		Percentage of total (n)	Percentage of total (n)	Percentage of total (n)	
Pathology	Lymphoplasmacytic infiltration	47.4 (27)	47.4 (27)	94.7 (54)	1.0000
	Fibrosis	33.3 (19)	35.1 (20)	68.4 (39)	0.9064
	Obliterative vasculitis	21.1 (12)	10.5 (6)	31.6 (18)	0.5852
	Has at least two features	38.6 (22)	33.3 (19)	71.9 (41)	0.7265
	Has at least three features	14 (8)	7 (4)	21.1 (12)	0.7292
	Eosinophilic infiltration	8.8 (5)	5.3 (3)	14.0 (8)	0.8615
	Has at least three features, including eosinophilia	17.5 (10)	8.8 (5)	26.3 (15)	0.6602
IgG4 count	Mean ± s.d. (*n*)	52±42 (24)	46±29 (22)	49±36 (46)	0.5792

*P*-values are indicated for two-sample *t*-tests between percentages for the highly likely and probable groups for each pathology category, and Student’s *t*-test for IgG4^+^ plasma cell count.

### Treatment outcomes

**Fig. 3:**
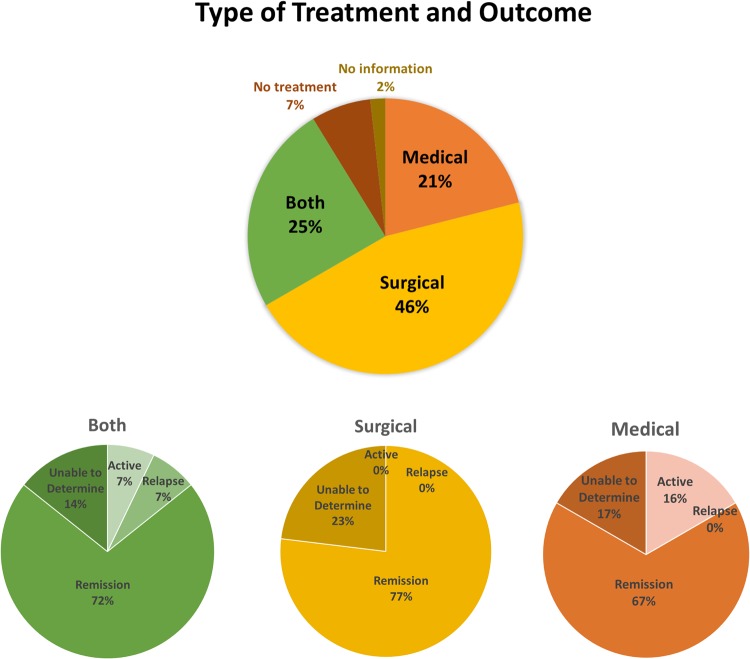
Type of treatment and outcome in our patient cohort Patients were designated as being in remission if indicated in the chart and/or symptom- and objective finding-free for >6 months, relapsed if symptoms/findings recurred after remission, active if no remission was achieved during follow-up, and as unable to determine if the duration of follow-up was <60 days or patients were lost to follow-up.

Almost half of the patients (45.6%; *n* = 26) were managed surgically, 21.1% (*n* = 12) medically, and 24.6% (*n* = 14) received both types of treatments. Medical treatment included prednisone (45.6%), MTX (5.3%), AZA (7%) and rituximab (8.8%); 58.3% of patients took more than one medication. Remission was achieved by 77% of surgical, 67% of medical, and 72% of recipients of both treatments (Fig. 3). Two of the five patients treated with rituximab achieved remission during the follow-up period of an average of 2.39 (0.33–6.46) years (Table 3). The surgical cohort included mostly organ types that were amenable to excision, such as the pancreas (*n* = 6), aorta (*n* = 3), biliary ducts (*n* = 4), salivary glands (*n* = 3), thyroid glands (*n* = 3), lacrimal glands (*n* = 1), lungs (*n* = 1), lymph node (*n* = 1) and other (*n* = 4, including buccal mass, terminal ileum, breast, liver and gallbladder).

**Table 3:** Treatment type and outcome by percentage of total patient population (*n* = 57) and the number of cases indicated in parentheses

	Medical	Surgical	Both	No treatment	No information
	**Percentage of total (*n*)**	**Percentage of total (*n*)**	**Percentage of total (*n*)**	**Percentage**	**Percentage**
Total	21.1 (12)	45.6 (26)	24.6 (14)	7.0 (4)	1.8 (1)
	**Percentage of medical (*n*)**	**Percentage of surgical (*n*)**	**Percentage of both (*n*)**	***P*-value**	
** **Prednisone	100 (12)	0 (0)	100 (14)		
** **MTX	8.3 (1)	0 (0)	14.3 (2)		
** **AZA	16.7 (2)	0 (0)	14.3 (2)		
** **Active	16.7 (2)	0.0 (0)	7.1 (1)	0.2962	
Relapse	0.0 (0)	0.0 (0)	7.1 (1)		
Remission	66.7 (8)	76.9 (20)	71.4 (10)		
Unable to determine	16.7 (2)	23.1 (6)	14.3 (2)		
Rituximab (*n*=5)	25 (*n* = 3 out of 12)	–	14.3 (*n* = 2 out of 14)		
Average number of prior medications	1.7	–	2.0		
Rituximab outcome	2 active, 1 unable to determine	–	2 remission		

The *P*-value was calculated for treatment outcome by the χ^2^ test. Percentages are indicated from the per-column total, with the number of patients in parentheses, except for total number of medical, surgical or both, no treatment and no information groups, where the percentages were calculated from the entire patient population of *n* = 57. The average number of prior medications for rituximab is the average number of medications patients took before being started on rituximab.

## Discussion

Herein, we present a single-centre analysis of IgG4-RD patient demographics, histopathological characteristics, organ involvement, treatment and outcomes spanning 10 years, using a patient population primarily identified based on tissue IgG4 plasma cell staining and histopathology. IgG4-RD is a rare disease, with an estimated prevalence of 6 per 100 000 and incidence of 0.28–1.08 cases per 100 000 inhabitants, respectively [20]. However, more than half of the cases reported in the literature have been Japanese [21], possibly because the disease was first identified in the Japanese population and therefore it is better recognized in this group [6, 21]. In our cohort, we identified 57 cases from 1481 pathology reports of positive IgG4 staining, from 1101 patients; thus, we report a prevalence of 5.2% of suspected cases. There is currently no literature on the actual prevalence of IgG4-RD out of suspected cases, and often no uniformity among studies of the original pool of patients from which the cases were identified. Examples include 235 cases identified from the radiology reports [19], an analysis of 100 consecutive cases from multiple centres in the UK [22], a study of 125 patients from Massachusetts General Hospital [23], and identification of 23 patients with tubulointerstitial nephritis out of 153 patients with suspected IgG4-RD [24]. In these studies, the diagnosis of IgG4-RD was also based on the pathology consensus statement, but they do not mention the proportion of patients ultimately identified as true IgG4-RD cases out of the original suspected cohort to enable comparison of the prevalence within each institution.

Likewise, it is important to note that only about half of the cases (*n* = 57) who met the IgG4 plasma cell staining criteria (*n* = 119) satisfied the IgG4-RD histopathological criteria, with 21.1% (*n* = 12) displaying all three characteristics of lymphoplasmacytic infiltration, fibrosis and obliterative phlebitis. Elevated IgG4 serum levels have recently been shown in a meta-analysis to have a pooled sensitivity of 85% and specificity of 93% [25], but the use of serum IgG4 levels for classification remains equivocal. For example, in one study only 51% of patients with active disease had elevated serum IgG4 levels (>135 mg/dl) [23]. In our cohort, only 57% (4 out of 7) of patients who had serum IgG4 >135 mg/dl demonstrated at least two histological features required for classification as highly likely. IgG4^+^ plasma cells can be increased in tissue biopsies from a number of other diseases, such as granulomatosis with polyangiitis, multicentric Castleman disease, Rosai–Dorfman disease, marginal zone lymphoma, pancreatic adenocarcinoma [26] and even valvular diseases [27]. Likewise, serum IgG4 can also be increased in chronic sinusitis, pneumonia, other autoimmune diseases and in some malignancies [26], indicating that sole reliance on IgG4 tissue staining or serum levels for diagnosis or classification of IgG4-RD can be misleading.

Also, the distribution of IgG4-RD histopathological findings is not well described in the literature. Many studies report serum IgG4 levels or IgG4^+^ plasma cell counts [22, 27, 28] and/or radiological findings [19, 27], but without clearly describing how the histopathology was analysed to deem a sample biopsy proven, other than referring to previous guidelines. These results underline the fact that a definitive diagnosis of IgG4-RD, a condition that can mimic various malignant, infectious and inflammatory conditions, remains difficult to make [8, 9] and that IgG4 serum levels and IgG4^+^ plasma cell counts have to be interpreted in the clinical and histopathological context [6, 26].

Our cohort had male-to-female ratio of 2:1, and a history of allergies was reported by 42% of cases, falling in the middle of previously reported ranges of 1.6–4:1 and 20–61.8%, respectively [28]. The ethnic distribution in our study was heterogeneous, with many fewer Asian patients (1.8%) compared with most studies from Japan, and fewer Caucasian patients (57.9%) than in a large cohort of 125 patients from Massachusetts General Hospital, in which 76% of patients were Caucasians [23]. With regard to the organs involved, the pancreas was the most affected organ in our cohort (26.4%) (Fig. 2A). This pancreatic predominance in our sample might well be the result of selection bias, because type 1 autoimmune pancreatitis is the most studied and recognized form of IgG4-RD [29, 30], and clinicians are more likely to consider IgG4-RD in the differential diagnosis of patients with pancreatic complaints. Other organs included a vast array, such as pericardium, liver, gallbladder, thymus, terminal ileum, appendix, rectum, left buccal mass, mesentery, breast, periureteral tissue and left renal mass. This is in agreement with other observations showing that IgG4-RD can affect essentially any organ, and the list of organs reported is growing [10, 31].

It was previously stated that the surgical option does not have an important role for treating IgG4-RD [28, 32], possibly because the surgical approach to certain affected tissues, such as the retroperitoneum, is difficult [32]. In our study, remission was achieved by 78.9% of surgery-only and both surgical and medical treatment. Our finding suggests that surgical intervention might be an effective treatment option in some cases of IgG4-RD that involve excisable tissue types. This finding is supported by another recently published study looking at treatment outcomes of 32 IgG4-RD patients with various organ manifestations [33]. The authors showed that 7 out of 32 patients who underwent primarily surgical intervention (2 surgery only, 3 surgery with glucocorticoids, and 1 surgery with glucocorticoid and MTX) achieved remission without recurrence after long-term follow-up [33]; the organs affected and subjected to surgical resection were the lungs, orbital tissue, thyroid and pericardium. Although we cannot draw definitive conclusions on which type of treatment should be the first line for patients with IgG4-RD, we found that 67% of medically treated patients achieved remission compared with ∼77% of those treated surgically. A χ^2^ test of different treatment outcomes between medicine-only, surgery-only and both types of treatments was not significant in our study (*P = *0.2962, Table 3), but this might be attributable to small sample size. Surgical treatment may spare patients the negative effects of long-term use of immunosuppressive drugs and may, in some cases, allow the complete removal of the mass. Although glucocorticoids are the recommended first-line treatment for IgG4-RD [34], >33% of patients relapse while receiving glucocorticoids, ≤64% relapse after cessation of glucocorticoids, and almost all (92%) relapse by 3 years of follow-up [23, 35, 36]. There are also significant side effects of long-term glucocorticoid use. In one study, for example, 27% of IgG4-RD patients treated with CSs developed new-onset diabetes or exacerbation of existing diabetes [37]. Diabetes mellitus is a common complication of type 1 autoimmune pancreatitis and occurs in approximately half of patients [30], and addition of long-term glucocorticoids can worsen diabetes. Therefore, especially in older, overweight and diabetic patients with IgG4-RD, use of glucocorticoids is not a good long-term solution. It is therefore necessary to design prospective studies to investigate the optimal treatment strategies for specific organ types of IgG4-RD, such as other drugs with fewer side effects and/or surgical removal for feasible tissue types.

There is also emerging evidence that rituximab, an anti-CD20 monoclonal antibody, might be an effective treatment in IgG4-RD [37–39]. Open-label rituximab use in IgG4-RD has recently undergone phase I and II trials with 30 patients, with complete remission of 46.7% at 6 months and 60% remission at any time point [40]. Of the five patients in our study treated with rituximab, two patients who also received two different prior medications and surgical intervention achieved remission by 6 months (Table 3). However, three patients who received rituximab as part of medical-only treatment after an average of 1.7 different prior medications were either still undergoing treatment or the outcome could not be determined at the time of data collection (Table 3).

Our study suffers from the inherent limitations of a retrospective design. As a relatively new disease, with evolving guidelines, identification of IgG4-RD patients relies on clinician suspicion as part of differential diagnosis, which biases our sample population to patients whose physicians were familiar enough with the disease to request the biopsy or staining for IgG4 plasma cells, and to patients who developed a mass in sites amenable to biopsy. Also, although a 6 month cut-off for remission induction stage is commonly used, as illustrated by a recent prospective study comparing glucocorticoids only *vs* glucocorticoid with immunosuppressive agent for IgG4-RD stage [41], it might be too short a period of time to gauge a significant clinical outcome to treatment. Also, assessment of remission may be difficult in some patients with IgG4-RD, a condition characterized by a fibro-inflammatory infiltrate, in whom, despite resolution of the inflammatory component, there is residual fibrotic tissue.

Moreover, as a systemic disease, IgG4-RD tends to involve multiple organs [28], which makes determination of the extent of disease difficult. In our cohort, we were restricted to the organ indicated by histopathological analysis as affected, and in most cases there was insufficient information about additional organs to determine the state of IgG4-RD definitely, although relapses in alternative sites other than the original organ affected by pathology were noted. Although the present study might not have captured the full extent of disease manifestation by IgG4-RD, it allowed us to track the outcomes of specific treatment for the primary organ involved.

In conclusion, IgG4-RD is a complex, systemic fibro-inflammatory condition that can affect virtually any organ system, with a prevalence of 5.2% of suspected cases. IgG staining criteria alone, in the absence of the other histopathological findings, can be misleading. We found that only about half of the cases (*n* = 57) who who had IgG4^+^ plasma cells ≥ 10/hpf or IgG4^+^/IgG^+^ ratio >40% (*n* = 119) satisfied the IgG4-RD histopathological criteria. In our study, although not statistically significant, surgical intervention resulted in a higher rate of remission than medical intervention. Patients treated surgically had a better remission rate by 6 months or a longer period of treatment than those with medical-only or both medical and surgical treatments. Our study illustrates the difficulty of making a precise diagnosis of IgG4 RD and the need for prospective studies that can result in better diagnosis and classification criteria and in better treatment strategies.

## Author contributions

All authors were involved in drafting the article or revising it critically for important intellectual content, and all authors approved the final version to be published. Drs. Lee and Gota had full access to all of the data in the study and take responsibility for the integrity of the data and the accuracy of the data analysis.

**Study conception and design.** Lee, Prayson, Gota

**Acquisition of data.** Lee, Alalwani, Prayson

**Analysis and interpretation of data.** Lee, Alalwani, Prayson, Gota

*Disclosure statement*: None of the authors have financial interests that could implicate potential conflict of interest.

*Funding*: This work was supported by Medical and Graduate Student Preceptorship from the Rheumatology Research Foundation (RRF) to Chan Mi Lee and Carmen E. Gota.
